# Investigating the patterns and determinants of seasonal variation in vitamin D status in Australian adults: the Seasonal D Cohort Study

**DOI:** 10.1186/s12889-016-3582-z

**Published:** 2016-08-26

**Authors:** Laura King, Keith Dear, Simone L. Harrison, Ingrid van der Mei, Alison M. Brodie, Michael G. Kimlin, Robyn M. Lucas

**Affiliations:** 1 National Centre for Epidemiology and Population Health, The Australian National University, Canberra, Australia; 2 Duke Kunshan University, Kunshan, China; 3 College of Public Health, Medical & Veterinary Sciences, James Cook, University, Townsville, Australia; 4 Menzies Research Institute Tasmania, University of Tasmania, Hobart, Australia; 5 Health Research Institute, University of the Sunshine Coast, Sippy Downs, Australia; 6 Queensland University of Technology, Brisbane, Australia

**Keywords:** Vitamin D, Cohort, Season, Australia, Adult, Determinants

## Abstract

**Background:**

Vitamin D status generally varies seasonally with changing solar UVB radiation, time in the sun, amount of skin exposed, and, possibly, diet. The Seasonal D Study was designed to quantify the amplitude and phase of seasonal variation in the serum concentration of 25-hydroxyvitamin D, (25OH)D)) and identify the determinants of the amplitude and phase and those of inter-individual variability in seasonal pattern.

**Methods:**

The Seasonal D Study collected data 2-monthly for 12 months, including demographics, personal sun exposure using a diary and polysulphone dosimeters over 7 days, and blood for serum 25(OH)D concentration. The study recruited 333 adults aged 18–79 years living in Canberra (35°S, *n* = 168) and Brisbane (27°South, *n* = 165), Australia.

**Discussion:**

We report the study design and cohort description for the Seasonal D Study. The study has collected a wealth of data to examine inter- and intra-individual seasonal variation in vitamin D status and serum 25(OH)D levels in Australian adults.

**Electronic supplementary material:**

The online version of this article (doi:10.1186/s12889-016-3582-z) contains supplementary material, which is available to authorized users.

## Background

Vitamin D is a fat soluble steroid pre-hormone that is produced endogenously following exposure of the skin to solar ultraviolet (UV) radiation. In many regions of the world, including Australia, this endogenous synthesis is the major source of vitamin D, with only a small proportion deriving from exogenous sources such as diet, fortified foods and supplements [1].

**Fig. 1:**
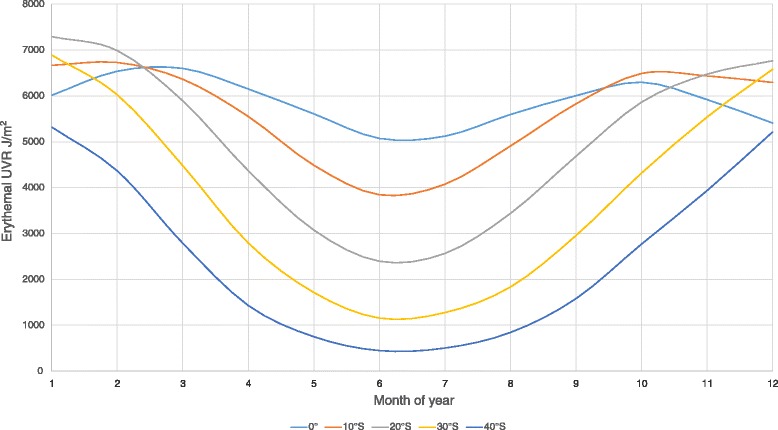
Variation in ambient UV radiation according to southerly latitude (in degrees) across the months of 2003 (data on ambient UV radiation from the TOMS satellite [18])

The amount of UV radiation that is present at Earth’s surface, and the wavelength composition, varies according to distance from the Equator (latitude), the hemisphere [2], the time of day, and the time of year. That is, UV irradiance is higher closer to the Equator (lower latitude), at solar noon, and in mid-summer when the sun is most directly overhead. In addition, seasonal variation in UV radiation has a greater amplitude with increasing latitude (Fig. 1).

It is generally accepted that only shorter wavelength UVB radiation initiates vitamin D synthesis [3]. UVB photons are absorbed by the vitamin D precursor, 7-dehydrocholesterol (7-DHC), which is present in the lipid bilayer of the plasma membrane of epidermal keratinocytes and dermal fibroblasts [4]. This results in chemical rearrangement of 7-DHC and the formation of pre-vitamin D_3_, which is rapidly (within 2 h) converted to vitamin D_3_. The latter is released from the plasma membrane into the extracellular space, where is it adsorbed by vitamin D binding protein in the dermal capillary bed and introduced into the circulation. Vitamin D_3_ is metabolised in the liver to 25-hydroxyvitamin D [25(OH)D, the usual measure of vitamin D status], and then in the kidney (or in target tissues) to its biologically active form, 1,25-dihydroxyvitamin D_3_ (1,25(OH)D_3_) [4].

Based on the seasonal variation in ambient solar UVB radiation, seasonal variation in 25(OH)D levels, of greater amplitude at higher latitudes, is expected. But there are moderating effects, particularly the amount of time spent outdoors, clothing cover, and use of sun protection. For example, in very hot locations (typically low latitude), people may spend more time indoors during summer, while in cool locations, the warmer weather of summer leads people to spend more time outdoors [5, 6]. In addition, more skin is typically covered by clothing in the winter and this is particularly apparent at higher latitudes. In cross-sectional studies where participants have had a single blood draw that occurs at different times during the year for different participants, there is a clear seasonal pattern to the mean 25(OH)D level [1, 7]. Only a few studies have examined an individual’s 25(OH)D levels across more than one time period. In these studies, although winter and summer 25(OH)D levels were correlated (e.g. *r* = 0.46 in Japanese women [8]) or minimum and maximum 25(OH)D levels were (*r* = 0.68 in Swedish blood donors [9]), there was substantial inter-individual variation in the seasonal pattern. A similar study of 60 healthy free-living adults from southeast Queensland, Australia aged 18–87 years (70 % female) showed that 25(OH)D level increased in summer for most individuals; however, for some individuals, 25(OH)D levels *decreased* in summer (correlation of winter and summer 25(OH)D levels *r* = 0.38) [10]. A key missing component of the vitamin D and health story, recently identified from a review of studies in the United Kingdom [11], is to understand the determinants of seasonal variation in vitamin D status. Identifying what determines whether and how quickly people become vitamin D depleted during winter, and what drives the differences in the amplitude of seasonal variation between individuals, is critical to advancing knowledge in this area.

There are important reasons to better understand inter- and intra-individual seasonal changes in 25(OH)D levels. First, observational studies typically test the association between the result of a single 25(OH)D test against disease risk, statistically “adjusting” for the seasonal effect based on the mean seasonal variation in 25(OH)D levels of the whole sample. The aim is to achieve measures of 25(OH)D level that are as though all participants had their blood taken on the same day. In longitudinal studies, more than one previous blood sample may be used, but in comparing either intra-individually or inter-individually, adjustment for season is usually required, as logistic considerations result in samples being taken at different times of the year. However, if there is poor correlation between winter and summer 25(OH)D, as has been previously observed [10], then an adjustment based on an individual maintaining their relationship to the mean, may not be valid. Failure to account adequately for inter-individual variation in seasonal pattern may result in spurious findings. Second, the degree of seasonal variation in 25(OH)D level itself may be a determinant of health. Increased disease risks at higher latitude may not be directly related to 25(OH)D level per se, but to the duration that a person remains vitamin D deficient in any given year, following winter (when ambient UV-B is at its lowest and clothing coverage is maximal). This possibility is difficult to investigate. Finally, understanding the amplitude and period of seasonal variation and their determinants may allow prediction of who is at risk of wintertime vitamin D deficiency based on demographic factors and 25(OH)D level measured in a different season. For example, the end of summer 25(OH)D level may allow prediction of the need for supplementation to avoid winter/early spring deficiency.

Here we report the study design and cohort description for the Seasonal D Study. This is a two-centre cohort study in which participants are tracked for 12 months, in order to assess and quantify the contribution of phenotypic, behavioural and environmental factors to inter- and intra-individual seasonal variation in vitamin D status. The novelty of the present study lies in the regular objective measurement of sun exposure and skin phototype, with two-monthly standardised measurements across two locations within Australia with very different climates.

## Methods

The Seasonal D Study was conducted in Canberra (35.3°S) which has a temperate climate with a marked summer/winter difference in temperature and ambient UV radiation; and Brisbane (27.5°S) which has a sub-tropical climate that remains relatively warm and humid year-round (Table 1).

**Table 1:** Location, Temperature, Rainfall and UV Radiation for 2013–4 in the two Seasonal D Study Regions, Canberra and Brisbane, Australia (Source: Australian Bureau of Meteorology www.bom.gov.au, and Australian Radiation and Nuclear Safety Agency, www.arpansa.gov.au)

	Latitude	Longitude	Average noon clear sky UV Index		Average Temperature °C		Average Rainfall (mm)	
			Summer (Jan)	Winter (July)	Summer (Jan)	Winter (July)	Summer (Jan)	Winter (July)
Brisbane	27.5 °S	153°E	10.7	3.8	30.6	22.1	212.7	26.6
Canberra	35.3°E	149°E	11.5	2.2	31.9	12.8	38.7	29.5

### Recruitment

The Seasonal D Study aimed to recruit 170 adult participants (18+ years) at each site with equal numbers of men and women and evenly spread across eleven age bands (18–24 years, then 5-yearly intervals between 25 and 75 years).

Recruitment commenced in both locations in September 2012, and was completed in September 2013. The cohort was initially drawn from a pool of participants from the AusD Study [12] who had indicated that they would be willing to participate in follow-up studies. To reach the final recruitment targets, the remaining participants were recruited through advertisements placed around workplaces, through word of mouth and through snowball recruiting. We did not aim to recruit a random sample of the population, rather a study sample with sufficient variability in lifestyles and demographics to support investigation of the influences on seasonal variation in vitamin D status.

Individuals who signalled an interest in the study were telephoned by a member of the research team to determine their eligibility and to obtain verbal consent. Individuals were eligible if they: were aged 18 years or older; had good comprehension of English; agreed to abstain from taking vitamin D supplements, multivitamins containing over 400 International Units (IU) of vitamin D, or cod liver oil, for the duration of their participation in the study; did not have a bleeding disorder; were not positive for Hepatitis B or C, or HIV; and were able to attend two-monthly on-site data collection interviews.

### Data collection

The initial data collection interview was scheduled 10–14 days after confirmation of eligibility and verbal agreement to participate. Once the interview was scheduled, each participant was mailed a package containing materials to be used for detailed measurement of their sun exposure over the 7 days prior to their interview, to include both working and non-working days. The package included: a) seven individually packaged and coded UV-sensitive polysulphone dosimeters (with wristband) for measurement of cumulative daily personal sun exposure, and b) a self-administered diary (see Additional file 1) in which to record daily sun exposure, physical activity and sun-protection (amount and type of clothing, sunscreen and shade utilization) over the 7 days of dosimetry [12, 13]. Polysulphone dosimeters have been used extensively to assess personal UVB (vitamin D effective) exposure, including in our previous AusD study [12, 14]. In the present study, dosimeters were attached to the left wrist using the wristband supplied, and were replaced daily to avoid reaching saturation. Detailed instructions on the use of dosimeters and sun diary were included in the package, to supplement the information provided during the telephone interview. A reminder SMS was sent to participants on the day before they were to commence wearing the dosimeters and completing the sun diary.

At the face-to-face interview, scheduled 1–2 days after completing 7-days of dosimetry, a research officer checked the completeness of the dosimeter and diary data, resolving any anomalies with the participant. Participants completed an online self-administered questionnaire (see Additional file 1) providing data on: date and place of birth, ancestry, current pregnancy and breastfeeding status, highest educational achievement, occupation held for the longest period, current employment status and occupation, total household income, smoking history, alcohol intake, self-rated health, current and past diagnosis of specified illnesses (including skin cancers and bone diseases), sun sensitivity (also used to assess Fitzpatrick skin type [15]), usual time spent outdoors recorded in hourly intervals for each day of the week during the previous month and usual use of sun protection when outdoors, dietary intake relevant to vitamin D, physical activity in the last 7 days [16], and usual time spent outdoors on working days and non-working days in each season. The full self-administered questionnaire was used for the baseline and end-of-study data collection, with a briefer questionnaire on factors that are likely to change over time, such as diet, for the interim data collection. Participants were also asked to bring their nutritional supplements and medications with them to the interview so that name and dose could be accurately recorded. Measurements were taken using standardised protocols, and included: height (baseline only); weight; waist and hip circumference; spectrophotometric skin reflectance (using a Minolta 2500d to record L*a*b values) on a sun-exposed body-site (the dorsum of the hand and the cheek) to record facultative skin colour and a sun-protected body-site (the upper inner arm) to record natural skin colour; blood pressure; and handgrip strength (using a portable dynamometer). A non-fasting blood sample was taken by venepuncture into a serum separator tube and, within four hours, the serum was removed and placed in 1 ml aliquots in cryotubes and frozen at −80 °C.

All participants undertook the same data collection protocol (7 days of UV dosimetry and sun diary recording, followed by self-administered questionnaire, measurements and blood collection at interview) every two months over a 12-month period (i.e. 7 data collection episodes). Data collection was completed in July 2014.

At the completion of the study, total serum 25(OH)D was measured using a Diasorin Liaison semi-automated chemiluminescence assay, with every tenth sample also measured by high performance liquid chromatography HPLC for quality assurance. This Diasorin Liaison assay at the Queensland University of Technology (QUT) participates in and is certified by the international Vitamin D External Quality Assessment Scheme. The inter- and intra-assay coefficients for this assay were 3–6 % and 6–9 %, respectively.

### Data management

Data on meteorological variables were available from routine measurements from instruments located at the nearby Canberra and Brisbane airports: ambient UV radiation levels from the Australian Radiation Protection and Nuclear Safety Agency monitors and weather variables (i.e. temperature, precipitation) from the Australian Bureau of Meteorology.

Questionnaire responses were entered directly into an online survey software tool (KeySurvey) then imported into Excel; all other data were scanned directly into an Access database. Dubious data were resolved by reference to the original forms. Diary days are excluded from the analysis if they had been insufficiently completed (i.e. the participant left the diary day blank), totalling 282 diary days. Sun diaries that did not have seven complete days recorded are retained in the analysis if there were at least three working days and at least one non-working day.

The absorbance at 330 nm of UV dosimeters was measured prior to, and following their use, with a spectrophotometer based at QUT; daily UV radiation exposure was calculated based on the change in pre-to-post absorbance, with a seasonal calibration factor applied. Results were reported as Standard Erythemal Dose (SED; 100 J/m^2^) [17].

### Statistical analysis

In this paper we provide a brief description of the study sample and the data available for analysis.

The Seasonal D Study was powered to analyse the characteristics of individually fitted seasonal curves for the variation in 25(OH)D levels, specifically their mean, amplitude and phase. We assumed that inter-individual variation would dominate the intra-individual error of estimation, and focused on modelling the mean 25(OH)D level as a function of the ambient UV radiation. The main factors influencing the study’s exposure variance, which in turn determines study power, were assumed to be location and season. On the log_10_ scale of UV Index, from available environmental data, the standard deviation (SD) across sites and seasons was estimated to be 0.345 log units. This approximates the study SD because there are equal numbers in each location and across time. We further assumed an error SD (in the estimated individual mean 25(OH)D level) of 25 nmol/L. The sample size to have good statistical power (90 % at *p* <0.05) was estimated to be *n* = 170, to detect a regression slope (beta) of 5 nmol/L or greater, per doubling of ambient UV radiation. As modelling the amplitude and phase the seasonal curve for 25(OH)D was likely to be less precise than modelling the mean, we represented this by a doubling of the error variance, requiring twice the sample size, or *N* = 340.

Data on all participants with more than one data collection time point will be included in the analysis. Data from the diary and UV dosimeters will be included if there are data for at least two working days, and one non-working day, for any data collection period. Participants with missing data on other variables will be excluded from analyses that include those specific variables.

We used the STROBE guidelines for reporting of observational studies to report the protocol and study sample description for the Seasonal D Study (see Additional file 2).

## Study progress

The Seasonal D Study has recruited a total of 333 adult participants aged 18 to 79 years. This includes 168 participants from the Canberra study region and 165 participants from the Brisbane study region. Of 2124 sun diaries completed, 87 % were completed in full for the seven days (*n* = 1848); 8.5 % had 6 days complete (*n* = 180), and 4.5 % of diaries had five or fewer days complete (*n* = 96). Complete data for seven sun diaries is available for 255 participants.

## Discussion

How to achieve and maintain vitamin D sufficiency, however it is defined, is a highly topical and contentious issue in many countries. Most studies investigating vitamin D status have been cross-sectional surveys that examine the prevalence of vitamin D insufficiency and deficiency, or the determinants of vitamin D status at a single time point. Seasonal variation is commonly reported, but relates to the mean 25(OH)D level of the study group who are sampled at different times of the year [1, 7].

Data from the Seasonal D Study will contribute to understanding how 25(OH)D levels vary across the year in locations with very different climates, as well as the determinants of intra-individual variation and inter-individual differences in seasonal patterns of 25(OH)D depletion/repletion. We will characterise seasonal impacts on a range of lifestyle factors, including diet, indoor/outdoor activities, physical activity, clothing cover, and climatic factors (principally temperature and ambient UV radiation), and quantify their effects on 25(OH)D levels.

The Seasonal D Study was purpose-designed to analyse seasonal variation in 25(OH)D concentration and vitamin D status and their determinants. Strengths include that the study included two locations with different climates to provide considerable variability in ambient UV radiation and other seasonally-varying factors. All data collection tools and methods were standardised across the two centres and research officers were trained to use a Standard Operating Procedure. The assays of 25(OH)D concentration were completed in a single laboratory in a batched analysis at the completion of the study, with good intra- and inter-batch agreement. One limitation of the study is that cohort is older (median age 49 years) and women are over represented, relative to the Australian population. These factors limit the generalisability of the findings. Furthermore the study is set solely in Australia, a country with high levels of ambient UV radiation. While this provides the potential for high variability in levels of exposure to UV radiation (ranging from individuals who work indoors and have indoor pastimes to the outdoor worker in a high ambient UV setting), it may further limit the generalisablity of the results to other populations in less sunny locations.

Current recommendations specify maintaining circulating 25(OH)D levels above 50 nmol/L throughout the year, yet are based on studies in which 25(OH)D concentration is measured at a single time point, without knowledge of, or consideration for, what may be a natural cyclical pattern of variability in 25(OH)D level by season. The Seasonal D Study will describe the typical rhythm of vitamin D production over the year for adults living in different climatic conditions. We will better understand the profile of Australian adults who are vitamin D deficient/sufficient all year round and understand which characteristics of individuals contribute to greater (or lesser) duration of time replete (above 50 nmol/L).

Although many studies, in various scientific fields, have quantified seasonality (usually using “cosinor analysis”), few have explicitly investigated its determinants using statistical models. Those few that do have limited their explorations to determinants of the magnitude of seasonal variation, as summarised through statistics such as the Gini coefficient. We aim to model the detailed characteristics of seasonal patterns, and determine how many data points per person are needed to accurately estimate an individual’s seasonal vitamin D profile, so that the profile itself, not just a single 25(OH)D level, can be tested in relation to disease risks.

Here we have provided the rationale, design, and methods for the Seasonal D Study which has followed 333 Australian adults over 12 months, with data collection on 7 occasions separated by approximately 2 months, providing detailed measurement of personal sun exposure and sun protection in relation to measured vitamin D status. Future work using these data will be to better understand the determinants of seasonal variation across the whole cohort, inter-individual variability within the cohort, and define intra-individual trajectories to support evidence-based sun protection and vitamin D maintenance recommendations.

## Additional files

Additional file 1: Questionnaires and sun diary for the Seasonal D Study. (PDF 482 kb) Additional file 2: Reporting against the STROBE guidelines. (DOC 101 kb)

## Abbreviations

1,25(OH)D_3_: 1,25-dihydroxyvitamin D_3_
25(OH)D: 25-hydroxyvitamin D
IU: International units
QUT: Queensland University of Technology
SED: Standard Erythemal Dose
SMS: Short message service
UV: Ultraviolet
UVB: Ultraviolet radiation in the UVB wavelengths (280 nm to 315 nm)
