# Supplementary material for: Investigating the patterns and determinants of seasonal variation in vitamin D status in Australian adults: the Seasonal D Cohort Study
Source: BMC Public Health. 2016 Aug 26;16(1):892. doi: 10.1186/s12889-016-3582-z (PMC5002142; doi:10.1186/s12889-016-3582-z)
Supplement: Additional file 1: — Questionnaires and sun diary for the Seasonal D Study. (PDF 482 kb) [file 12889_2016_3582_MOESM1_ESM.pdf]

DAY 4

Date ☐☐ / ☐☐ / ☐☐☐☐ **Participant ID:** ☐☐☐☐

UV Badge Number: ☐☐☐☐☐ **Interview Number:** ☐

	TIME OUTDOORS IN THE SUN (Cross the box which best represents the amount of time that you spent in the sun during each one hour interval shown below)					Did you apply sun-screen?	Where did you apply sunscreen?	SPF of Sunscreen	TYPE OF CLOTHING WORN Please refer to Clothing Guide (You must write one number in each box)					LEVEL OF PHYSICAL ACTIVITY 0 = None 1 = Mild 2 = Moderate 3 = Vigorous (You must write one number in each box)	
	0 mins	<15 mins	15-29 mins	30-44 mins	45-60 mins	Cross all that apply Leave blank if not used	1=ALL exposed body sites 2=MORE THAN HALF of exposed body sites 3=LESS THAN HALF of exposed body sites	(number only, omit '+')	Upper body	Lower body	Head wear	Foot-wear	Gloves (Cross all that apply) Leave blank if not used		
**Morning**														Indoors	Outdoors
5 - 6 am	☐	☐	☐	☐	☐	☐	☐	☐☐	☐	☐	☐	☐	☐	☐	☐
6 - 7 am	☐	☐	☐	☐	☐	☐	☐	☐☐	☐	☐	☐	☐	☐	☐	☐
7 - 8 am	☐	☐	☐	☐	☐	☐	☐	☐☐	☐	☐	☐	☐	☐	☐	☐
8 - 9 am	☐	☐	☐	☐	☐	☐	☐	☐☐	☐	☐	☐	☐	☐	☐	☐
9 - 10 am	☐	☐	☐	☐	☐	☐	☐	☐☐	☐	☐	☐	☐	☐	☐	☐
10 - 11 am	☐	☐	☐	☐	☐	☐	☐	☐☐	☐	☐	☐	☐	☐	☐	☐
11 - 12 am	☐	☐	☐	☐	☐	☐	☐	☐☐	☐	☐	☐	☐	☐	☐	☐
**Afternoon**															
12 - 1 pm	☐	☐	☐	☐	☐	☐	☐	☐☐	☐	☐	☐	☐	☐	☐	☐
1 - 2 pm	☐	☐	☐	☐	☐	☐	☐	☐☐	☐	☐	☐	☐	☐	☐	☐
2 - 3 pm	☐	☐	☐	☐	☐	☐	☐	☐☐	☐	☐	☐	☐	☐	☐	☐
3 - 4 pm	☐	☐	☐	☐	☐	☐	☐	☐☐	☐	☐	☐	☐	☐	☐	☐
4 - 5 pm	☐	☐	☐	☐	☐	☐	☐	☐☐	☐	☐	☐	☐	☐	☐	☐
5 - 6 pm	☐	☐	☐	☐	☐	☐	☐	☐☐	☐	☐	☐	☐	☐	☐	☐
6 - 7 pm	☐	☐	☐	☐	☐	☐	☐	☐☐	☐	☐	☐	☐	☐	☐	☐

Did you make sure that your wristband was not covered by clothing today? ☐ **Yes** ☐ **No**

Please document any problems you had with the UV badge (e.g. The badge was covered by clothing for approx 30 minutes while I was outside at midday; the badge fell off at 3pm; the badge got wet etc)

General Health and Sun Exposure Questionnaire

Thank you for agreeing to take part in this study. We are conducting this study to see how vitamin D levels in a person change throughout the year, and to uncover what factors about that person or their environment, influence these changes in vitamin D levels. This questionnaire will help us to gather important information about things that affect your vitamin D levels.

Please answer the following questions the best that you can.

At the end of the study, once your vitamin D and UV badge results have been matched to this questionnaire, your name will be removed from our records.
All of the information that you provide will remain strictly confidential.

1. Participant ID

Please use the blank space to write your answers.

Participant ID

....

2. Place and date of birth:

Please use the blank space to write your answers.

City/Town of birth

....

Country of birth

....

If not Australia, in what year did you come to Australia?

....

Date of birth (dd/mm/yyyy)

....

3. In what state/territory do you live?

Please pick one of the answers below.

- Queensland
- Australian Capital Territory

4. How would you describe your ancestry?

Please pick one of the answers below.

- Australian Aboriginal
- North-West European (including British, Irish, Western European, Northern European)
- Southern European (including Spanish, Italian, Portuguese, etc)
- Torres Strait Islander
- New Zealander/Maori
- Melanesian and Papuan
- Micronesian
- Polynesian
- South-East Asian (including Burmese, Thai, Vietnamese, Indonesian, Malay etc)
- South Eastern European (including Albanian, Croatian, Greek, Cypriot etc)
- Eastern European (including Belarusian, Hungarian, Polish, Russian, Ukrainian etc)
- North African and Middle Eastern (including Algerian, Lebanese, Israeli, Turkish etc)
- North-East Asian (including Chinese, Japanese, Korean, Mongolian etc)
- Southern Asian (including Indian, Pakistani, Bangladeshi)
- Central Asian (including Afghan, Georgian, Kazakh, Turkmen etc)
- North American Aboriginal
- Central or South American
- Caribbean Islander
- Central, West, South and East African
- Mixed Race
- Other

5. Please select your gender

Please pick one of the answers below.

- Male Go to question 8
- Female

6. Are you currently pregnant?

Please pick one of the answers below.

- Yes
- No

7. Are you currently breastfeeding?

Please pick one of the answers below.

- Yes
- No

8. What is the highest technical, professional or academic qualification that you have completed?

Please pick one of the answers below.

- Did not complete primary school
- Primary school
- Some high school (Year 11 or under)
- Year 12 Senior Certificate (or HSC)
- Trade/Apprenticeship
- Certificate or Diploma
- Bachelor's Degree
- Postgraduate Degree

9. Which of the following best describes the occupation you had for the longest period?

Please pick one of the answers below or add your own.

- Manager
- Professional
- Technicians and Trades Workers
- Community and Personal Service Workers
- Clerical and Administrative Workers
- Sales Workers
- Machinery Operators and Drivers
- Labourer or related worker

Other

....

10. Which of the following best describes your current employment status?

Please pick one of the answers below or add your own.

- Unemployed
- Home duties
- Part-time work -employed/self-employed
- Full-time work - employed/self-employed
- Student
- Sole parent pension
- Disability pension
- Retired

Other

....

11. Which of the following best describes your current, main occupation?

Please pick one of the answers below.

- Mainly indoors (e.g. office worker)
- Half indoors and half outdoors (e.g. physical education teacher)
- Mainly outdoors (e.g. gardener)

12. Have you ever smoked?

Please pick one of the answers below.

- Yes
- No Go to question 16

13. Are you currently a regular smoker of cigarettes or cigars or pipes?

Please pick one of the answers below.

- Yes Go to question 15
- No

14. When did you quit smoking?

Please use the blank space to write your answers.

(dd/mm/yyyy) Go to question 16

....

15. Over the past month, on average, what number of the following items did you smoke per day?

Please use the blank space to write your answers.

Filter cigarettes

....

Hand rolled cigarettes

....

Pipefulls of tobacco

....

Cigars/cigarillos

....

Hookah/Shisha

....

16. Do you drink alcohol?

Please pick one of the answers below.

- ☐ Yes
- ☐ No Go to question 18

17. How many alcoholic drinks (not units) of each of the following do you have each week? Please put "0" if you have less than one drink each week.

Please use the blank space to write your answers.

Beer (285ml "Middy/Half Pint/Pot") Full Strength, 4.8% Alc. Vol

....

Beer (375 ml can) Full Strength, 4.8% Alc Vol

....

Beer (375 ml bottle or "Stubby") Full Strength 4.8% Alc Vol

....

Beer (425ml "Schooner") Full Strength, 4.8% Alc Vol

....

Wine, red (150 ml glass, average restaurant serving) 13.5% Alc Vol

....

Wine, white (150 ml glass, average restaurant serving) 11.5% Alc Vol

....

Wine, sparkling (150 ml glass, average restaurant serving) 12% Alc Vol

....

Spirit nip (30 ml) High strength, 40% Alc Vol

....

Spirits, pre-mixed (375 ml) Full Strength, 5% Alc Vol

....

Spirits, ready to drink bottle (330 ml) 5% Alc Vol

....

Total

....

18. In general, how would you rate your health?

Please pick one of the answers below.

- Excellent
- Very good
- Good
- Fair
- Poor

19. Has a doctor ever told you that you have any of the following medical conditions?

Please check all that apply.

- ☐ Osteoporosis or osteopaenia
- ☐ Diabetes
- ☐ High blood pressure (hypertension)
- ☐ High cholesterol (dyslipidaemia)
- ☐ Heart disease
- ☐ Cancer
- ☐ Kidney disease
- ☐ Liver disease
- ☐ Mental health problems
- ☐ I have not been told that I have any medical conditions

20. If you have been diagnosed with cancer, please tick which one(s) below.

Please check all that apply and/or add your own variant.

- ☐ Melanoma
- ☐ Other skin cancer
- ☐ Colorectal cancer
- ☐ Other gastrointestinal cancer (oesophageal, stomach, intestine)
- ☐ Breast cancer
- ☐ Prostate cancer
- ☐ Lung cancer
- ☐ Gynaecological cancer (cervical, uterine or ovarian)
- ☐ Lymphoma, leukaemia or Non-Hodgkin's Lymphoma

Other

....

21. What is your natural eye colour?

Please pick one of the answers below.

- Blue, light blue, grey blue, blue-green
- Green
- Hazel, green hazel
- Brown, dark brown

22. What is your natural hair colour (when you were 18 years old)?

Please pick one of the answers below.

- Red
- Blond
- Chestnut or dark blond
- Brown
- Black

23. What is your natural (untanned) skin colour (on non-sun exposed skin)?

Please pick one of the answers below.

- Fair
- Medium
- Olive
- Dark/Black

24. How many freckles do you have on unexposed areas?

Please pick one of the answers below.

- Many
- Several
- Few
- Rare
- None

25. How does your skin react if you were to sit in the sun in your current area of residence, in the middle of the day, for the first time in summer, without sunscreen?

Please pick one of the answers below.

- Never burn
- Burn after more than 2 hours sun exposure
- Burn after 1 - 2 hours
- Burn after 1/2 - 1 hour
- Burn within half an hour

26. How would your skin react if you were to sit in the sun in your current area of residence, for one hour in the middle of the day, for the first time in summer, without sunscreen?

Please pick one of the answers below.

- Burn then peel
- Burn then tan
- Tan only

27. At the end of the summer or after a two week holiday in the sun, what kind of tan would you have?

Please pick one of the answers below.

- A dark tan
- A medium tan
- A light tan
- Practically no tan

28. Have you worked a night shift at least once in the past month?

Please pick one of the answers below.

- Yes
- No Go to question 30

29. About how many night shifts have you worked in the past month?

Please pick one of the answers below.

- 1 night shift
- 2 night shifts
- 3 night shifts
- 4 night shifts
- 5-7 night shifts
- 8-10 night shifts
- 11-13 night shifts
- 14-16 night shifts
- 17-19 night shifts
- 20+ night shifts

30. We are interested in the CLOTHING AND SUN PROTECTION that you have worn in the PAST MONTH when you were outside. Di you wear or use...

Please mark the corresponding circle - only one per line.

	A	B	C	D	E	F
Hat, cap or other head covering?						
Long sleeves?						
Clothing that covers most of your legs?						
Sunglasses?						
Umbrella in the sun?						

Legend for rank grid table: 30. We are interested in the CLOTHING AND SUN PROTECTION that you have worn in the PAST MONTH when you were outside. Di you wear or use...

Columns:

A - Never
B - Rarely
C - Sometimes
D - Often
E - Almost always
F - Always

31. Have you used sunscreen in the PAST MONTH, including moisturiser and make-up with sun protection factor (SPF)?

Please pick one of the answers below.

- Yes
- No Go to question 36

32. What is the sun-protection factor (SPF) of the sunscreen that you have used most often? Consider any product you may use that have an SPF.

Please mark the corresponding circle - only one per line.

	SPF 15	SPF 30	SPF 50
Sunscreen			
Moisturiser			
Make-up			

33. Over the PAST MONTH, how often on average have you used any of the products with sunscreen you described in the previous question?

Please pick one of the answers below.

- Every day
- 5-7 days a week
- 3-4 days a week
- 1-2 days a week
- Less than once a week

34. On days that you have used sunscreen in the PAST MONTH, how many times per day did you apply it?

Please use the blank space to write your answers.

....

35. Please select each area of your body where you used sunscreen.

Please check all that apply.

- ☐ Face
- ☐ Head
- ☐ Neck
- ☐ Chest
- ☐ Upper arms
- ☐ Lower arms
- ☐ Upper legs
- ☐ Lower legs
- ☐ Hands
- ☐ Feet
- ☐ Back

36. Please check the number of serves that you have consumed for each food in the past month.

Please fill in the answers in the table below (mark appropriate circles and squares and fill in the blank spaces).

	Number of Serves	Daily	Weekly	Monthly
Fresh salmon (1 serve = 120 g cooked, or about palm size)	....			
Salmon, tinned (1 serve= 100 g)	....			
Fresh tuna (1 serve = 120 g cooked, or about palm size)	....			
Tinned tuna (1 serve = 100 g)	....			
Sardines (1 serve = 60 g, about 1/2 tin)	....			
Mackerel or Herring (1 serve = 100 g)	....			
Milk or yoghurt fortified with vitamin D (1 serve = 250 mL, 1 cup milk or yoghurt)	....			
Milk (1 serve = 250 ml or 1 cup)	....			
Yoghurt (1 serve= 125g-160 g)	....			
Cheese (1 serve = 40 g, 2 slices)	....			

PHYSICAL ACTIVITY The following questions will ask you about the time you spent being physically active in the last 7 days. Please answer each question even if you do not consider yourself to be an active person. Please think about the activities you do at work, as part of your house and yard work, to get from place to place, and in your spare time for recreation, exercise or sport. Driving does not count as physical activity.

37. Think about all the vigorous activities that you did in the last 7 days. Vigorous physical activities refer to activities that take hard physical effort and make you breathe much harder than normal. Think only about those physical activities you did for at least 10 minutes at a times such as heavy lifting, digging, aerobics, running, swimming, or fast bicycling.

Please fill in the answers in the table below (mark appropriate circles and squares and fill in the blank spaces).

	Days per week	Don't know/Not sure	No vigorous physical activities
During the last 7 days on how many days did you do vigorous physical activities?	....		

38. How much time (in minutes) did you usually spend doing vigorous physical activities on one of those days?

Please use the blank space to write your answers.

Inside (minutes per day)?

....

Outside (minutes per day)?

....

39. Think about all the moderate activities that you did in the last 7 days. Moderate physical activities refer to activities that take moderate physical effort and make you breathe somewhat harder than normal. Think only about those physical activities you did for at least 10 minutes at a times such as carrying light loads, bicycling at a heavy pace, slow jogging, or doubles tennis. Do not include walking.

Please fill in the answers in the table below (mark appropriate circles and squares and fill in the blank spaces).

	Days per week	Don't know/Not sure	No moderate physical activities
During the last 7 days on how many days did you do moderate physical activities?	....		

40. How much time (in minutes) did you usually spend doing moderate physical activities on one of those days?

Please use the blank space to write your answers.

Inside (minutes per day)?

....

Outside (minutes per day)?

....

41. Think about all the walking that you did in the last 7 days. This includes at work and at home, walking to travel from place to place, and walking that you might do solely for recreation, sport, exercise or leisure.

Please fill in the answers in the table below (mark appropriate circles and squares and fill in the blank spaces).

	Days per week	Don't know/Not sure	No walking
During the last 7 days on how many days did you walk for at least 10 minutes at a time?	....	☐	☐

42. How much time (IN MINUTES) did you usually spend on ONE OF THOSE DAYS....

Please use the blank space to write your answers.

Walking INSIDE (minutes per day)?

....

Walking OUTSIDE (minutes per day)?

....

Sitting INSIDE (minutes per day)?

....

Sitting OUTSIDE (minutes per day)?

....

43. Is the pattern of physical activity that you described above for the past 7 days, typical of your physical activity over the past month?

Please pick one of the answers below.

- ☐ Yes Go to question 45
- ☐ No

44. Do you usually spend:

Please pick one of the answers below.

- ☐ MORE time being physically active?
- ☐ LESS time being physically active?

45. In SUMMER (DECEMBER TO FEBRUARY) , what times of the day would you usually be outside, and how long would you be outside for on a typical WORKING DAY?

Please mark the corresponding circle - only one per line.

	Never	<15min	15-29min	30-44min	45-60min
5-6 am					
6-7 am					
7-8 am					
8-9 am					
9-10 am					
10-11 am					
11-12 pm					
12-1 pm					
1-2 pm					
2-3 pm					
3-4 pm					
4-5 pm					
5-6 pm					
6-7 pm					

46. In AUTUMN (MARCH TO MAY), would your pattern of time outside on a typical WORKING DAY be the same as it was on a typical working day in SUMMER?

Please pick one of the answers below.

- Yes Go to question 48
- No

47. In AUTUMN (MARCH TO MAY) , what times of the day would you usually be outside, and how long would you be outside for on a typical WORKING DAY?

Please mark the corresponding circle - only one per line.

	Never	<15min	15-29min	30-44min	45-60min
5-6 am					
6-7 am					
7-8 am					
8-9 am					
9-10 am					
10-11 am					
11-12 pm					
12-1 pm					
1-2 pm					
2-3 pm					
3-4 pm					
4-5 pm					
5-6 pm					
6-7 pm					

48. In WINTER (JUNE TO AUGUST), would your pattern of time outside on a typical WORKING DAY be the same as it was on a typical working day in SUMMER?

Please pick one of the answers below.

- Yes Go to question 50
- No

49. In WINTER (JUNE TO AUGUST) , what times of the day would you usually be outside, and how long would you be outside for on a typical WORKING DAY?

Please mark the corresponding circle - only one per line.

	Never	<15min	15-29min	30-44min	45-60min
5-6 am					
6-7 am					
7-8 am					
8-9 am					
9-10 am					
10-11 am					
11-12 pm					
12-1 pm					
1-2 pm					
2-3 pm					
3-4 pm					
4-5 pm					
5-6 pm					
6-7 pm					

50. In SPRING (SEPTEMBER TO NOVEMBER), would your pattern of time outside on a typical WORKING DAY be the same as it was on a typical working day in SUMMER?

Please pick one of the answers below.

- Yes Go to question 52
- No

51. In SPRING (SEPTEMBER TO NOVEMBER) , what times of the day would you usually be outside, and how long would you be outside for on a typical WORKING DAY?

Please mark the corresponding circle - only one per line.

	Never	<15min	15-29min	30-44min	45-60min
5-6 am					
6-7 am					
7-8 am					
8-9 am					
9-10 am					
10-11 am					
11-12 pm					
12-1 pm					
1-2 pm					
2-3 pm					
3-4 pm					
4-5 pm					
5-6 pm					
6-7 pm					

52. In SUMMER (DECEMBER TO FEBRUARY) , what times of the day would you usually be outside, and how long would you be outside for on a typical NON-WORKING DAY?

Please mark the corresponding circle - only one per line.

	Never	<15min	15-29min	30-44min	45-60min
5-6 am					
6-7 am					
7-8 am					
8-9 am					
9-10 am					
10-11 am					
11-12 pm					
12-1 pm					
1-2 pm					
2-3 pm					
3-4 pm					
4-5 pm					
5-6 pm					
6-7 pm					

53. In AUTUMN (MARCH TO MAY) , what times of the day would you usually be outside, and how long would you be outside for on a typical NON-WORKING DAY?

Please mark the corresponding circle - only one per line.

	Never	<15min	15-29min	30-44min	45-60min
5-6 am					
6-7 am					
7-8 am					
8-9 am					
9-10 am					
10-11 am					
11-12 pm					
12-1 pm					
1-2 pm					
2-3 pm					
3-4 pm					
4-5 pm					
5-6 pm					
6-7 pm					

54. In WINTER (JUNE TO AUGUST) , what times of the day would you usually be outside, and how long would you be outside for on a typical NON-WORKING DAY?

Please mark the corresponding circle - only one per line.

	Never	<15min	15-29min	30-44min	45-60min
5-6 am					
6-7 am					
7-8 am					
8-9 am					
9-10 am					
10-11 am					
11-12 pm					
12-1 pm					
1-2 pm					
2-3 pm					
3-4 pm					
4-5 pm					
5-6 pm					
6-7 pm					

55. In SPRING (SEPTEMBER TO NOVEMBER) , what times of the day would you usually be outside, and how long would you be outside for on a typical NON-WORKING DAY?

Please mark the corresponding circle - only one per line.

	Never	<15min	15-29min	30-44min	45-60min
5-6 am					
6-7 am					
7-8 am					
8-9 am					
9-10 am					
10-11 am					
11-12 pm					
12-1 pm					
1-2 pm					
2-3 pm					
3-4 pm					
4-5 pm					
5-6 pm					
6-7 pm					

56. What is your total annual household income, before tax, from all sources (including pensions, benefits, superannuation)?

Please pick one of the answers below.

- Less than $10,000 per year
- $10,000 - $19,999
- $20,000 - $29,999
- $30,000 - $49,999
- $50,000 - $69,999
- $70,000 - $89,999
- $90,000 - $129,999
- $130,000 - $207,999
- $208,000 or more
- I would prefer not to answer this question

Print 2 to 6

Welcome back to your 2nd questionnaire for the Seasonal D study. Thanks for taking the time to complete these questions.

1. Please enter your Participant ID number below. If you do not remember, please ask the research officer.

Please use the blank space to write your answers.

Participant ID number:

....

2. Please check the number of serves that you have consumed for each food in the past month.

Please fill in the answers in the table below (mark appropriate circles and squares and fill in the blank spaces).

	Number of Serves	Daily	Weekly	Monthly
Fresh salmon (1 serve = 120 g cooked, or about palm size)	....	○	○	○
Salmon, tinned (1 serve= 100 g)	....	○	○	○
Fresh tuna (1 serve = 120 g cooked, or about palm size)	....	○	○	○
Tinned tuna (1 serve = 100 g)	....	○	○	○
Sardines (1 serve = 60 g, about 1/2 tin)	....	○	○	○
Mackerel or Herring (1 serve = 100 g)	....	○	○	○
Milk or yoghurt fortified with vitamin D (1 serve = 250 mL, 1 cup milk or yoghurt)	....	○	○	○
Milk (1 serve = 250 ml or 1 cup)	....	○	○	○
Yoghurt (1 serve= 125g-160 g)	....	○	○	○
Cheese (1 serve = 40 g, 2 slices)	....	○	○	○

3. Is the pattern of physical activity that you described in your sun diary typical of your physical activity over the past month?

Please pick one of the answers below.

- ○ Yes
- ○ No

4. Are you usually more or less physically active?

Please pick one of the answers below.

- ○ More
- ○ Less

Thanks for your time!

The Seasonal D Research Team

Print 7

Welcome back to your 2nd questionnaire for the Seasonal D study. Thanks for taking the time to complete these questions.

1. Please enter your Participant ID number below. If you do not remember, please ask the research officer.

Please use the blank space to write your answers.

Participant ID number:

....

2. Please check the number of serves that you have consumed for each food in the past month.

Please fill in the answers in the table below (mark appropriate circles and squares and fill in the blank spaces).

	Number of Serves	Daily	Weekly	Monthly
Fresh salmon (1 serve = 120 g cooked, or about palm size)	....	○	○	○
Salmon, tinned (1 serve= 100 g)	....	○	○	○
Fresh tuna (1 serve = 120 g cooked, or about palm size)	....	○	○	○
Tinned tuna (1 serve = 100 g)	....	○	○	○
Sardines (1 serve = 60 g, about 1/2 tin)	....	○	○	○
Mackerel or Herring (1 serve = 100 g)	....	○	○	○
Milk or yoghurt fortified with vitamin D (1 serve = 250 mL, 1 cup milk or yoghurt)	....	○	○	○
Milk (1 serve = 250 ml or 1 cup)	....	○	○	○
Yoghurt (1 serve= 125g-160 g)	....	○	○	○
Cheese (1 serve = 40 g, 2 slices)	....	○	○	○

3. Is the pattern of physical activity that you described in your sun diary typical of your physical activity over the past month?

Please pick one of the answers below.

- O Yes Got to Question 5
- O No

4. Are you usually more or less physically active?

Please pick one of the answers below.

- O More
- O Less

5. In SUMMER (DECEMBER TO FEBRUARY) , what times of the day would you usually be outside, and how long would you be outside for on a typical WORKING DAY?

Please mark the corresponding circle - only one per line.

	Never	<15min	15-29min	30-44min	45-60min
5-6 am	O	O	O	O	O
6-7 am	O	O	O	O	O
7-8 am	O	O	O	O	O
8-9 am	O	O	O	O	O
9-10 am	O	O	O	O	O
10-11 am	O	O	O	O	O
11-12 pm	O	O	O	O	O
12-1 pm	O	O	O	O	O
1-2 pm	O	O	O	O	O
2-3 pm	O	O	O	O	O
3-4 pm	O	O	O	O	O
4-5 pm	O	O	O	O	O
5-6 pm	O	O	O	O	O
6-7 pm	O	O	O	O	O

6. In AUTUMN (MARCH TO MAY), would your pattern of time outside on a typical WORKING DAY be the same as it was on a typical working day in SUMMER?

Please pick one of the answers below.

O Yes Go to question 8

O No

7. In AUTUMN (MARCH TO MAY) , what times of the day would you usually be outside, and how long would you be outside for on a typical WORKING DAY?

Please mark the corresponding circle - only one per line.

	Never	<15min	15-29min	30-44min	45-60min
5-6 am	O	O	O	O	O
6-7 am	O	O	O	O	O
7-8 am	O	O	O	O	O
8-9 am	O	O	O	O	O
9-10 am	O	O	O	O	O
10-11 am	O	O	O	O	O
11-12 pm	O	O	O	O	O
12-1 pm	O	O	O	O	O
1-2 pm	O	O	O	O	O
2-3 pm	O	O	O	O	O
3-4 pm	O	O	O	O	O
4-5 pm	O	O	O	O	O
5-6 pm	O	O	O	O	O
6-7 pm	O	O	O	O	O

8. In WINTER (JUNE TO AUGUST), would your pattern of time outside on a typical WORKING DAY be the same as it was on a typical working day in SUMMER?

Please pick one of the answers below.

O Yes Go to question 10

O No

9. In WINTER (JUNE TO AUGUST) , what times of the day would you usually be outside, and how long would you be outside for on a typical WORKING DAY?

Please mark the corresponding circle - only one per line.

	Never	<15min	15-29min	30-44min	45-60min
5-6 am	○	○	○	○	○
6-7 am	○	○	○	○	○
7-8 am	○	○	○	○	○
8-9 am	○	○	○	○	○
9-10 am	○	○	○	○	○
10-11 am	○	○	○	○	○
11-12 pm	○	○	○	○	○
12-1 pm	○	○	○	○	○
1-2 pm	○	○	○	○	○
2-3 pm	○	○	○	○	○
3-4 pm	○	○	○	○	○
4-5 pm	○	○	○	○	○
5-6 pm	○	○	○	○	○
6-7 pm	○	○	○	○	○

10. In SPRING (SEPTEMBER TO NOVEMBER), would your pattern of time outside on a typical WORKING DAY be the same as it was on a typical working day in SUMMER?

Please pick one of the answers below.

○ Yes Go to question 12

○ No

11. In SPRING (SEPTEMBER TO NOVEMBER) , what times of the day would you usually be outside, and how long would you be outside for on a typical WORKING DAY?

Please mark the corresponding circle - only one per line.

	Never	<15min	15-29min	30-44min	45-60min
5-6 am	○	○	○	○	○
6-7 am	○	○	○	○	○
7-8 am	○	○	○	○	○
8-9 am	○	○	○	○	○
9-10 am	○	○	○	○	○
10-11 am	○	○	○	○	○
11-12 pm	○	○	○	○	○
12-1 pm	○	○	○	○	○
1-2 pm	○	○	○	○	○
2-3 pm	○	○	○	○	○
3-4 pm	○	○	○	○	○
4-5 pm	○	○	○	○	○
5-6 pm	○	○	○	○	○
6-7 pm	○	○	○	○	○

12. In SUMMER (DECEMBER TO FEBRUARY) , what times of the day would you usually be outside, and how long would you be outside for on a typical NON-WORKING DAY?

Please mark the corresponding circle - only one per line.

	Never	<15min	15-29min	30-44min	45-60min
5-6 am	○	○	○	○	○
6-7 am	○	○	○	○	○
7-8 am	○	○	○	○	○
8-9 am	○	○	○	○	○
9-10 am	○	○	○	○	○
10-11 am	○	○	○	○	○
11-12 pm	○	○	○	○	○
12-1 pm	○	○	○	○	○
1-2 pm	○	○	○	○	○
2-3 pm	○	○	○	○	○
3-4 pm	○	○	○	○	○
4-5 pm	○	○	○	○	○
5-6 pm	○	○	○	○	○
6-7 pm	○	○	○	○	○

13. In AUTUMN (MARCH TO MAY) , what times of the day would you usually be outside, and how long would you be outside for on a typical NON-WORKING DAY?

Please mark the corresponding circle - only one per line.

	Never	<15min	15-29min	30-44min	45-60min
5-6 am	○	○	○	○	○
6-7 am	○	○	○	○	○
7-8 am	○	○	○	○	○
8-9 am	○	○	○	○	○
9-10 am	○	○	○	○	○
10-11 am	○	○	○	○	○
11-12 pm	○	○	○	○	○
12-1 pm	○	○	○	○	○
1-2 pm	○	○	○	○	○
2-3 pm	○	○	○	○	○
3-4 pm	○	○	○	○	○
4-5 pm	○	○	○	○	○
5-6 pm	○	○	○	○	○
6-7 pm	○	○	○	○	○

14. In WINTER (JUNE TO AUGUST) , what times of the day would you usually be outside, and how long would you be outside for on a typical NON-WORKING DAY?

Please mark the corresponding circle - only one per line.

	Never	<15min	15-29min	30-44min	45-60min
5-6 am	○	○	○	○	○
6-7 am	○	○	○	○	○
7-8 am	○	○	○	○	○
8-9 am	○	○	○	○	○
9-10 am	○	○	○	○	○
10-11 am	○	○	○	○	○
11-12 pm	○	○	○	○	○
12-1 pm	○	○	○	○	○
1-2 pm	○	○	○	○	○
2-3 pm	○	○	○	○	○
3-4 pm	○	○	○	○	○
4-5 pm	○	○	○	○	○
5-6 pm	○	○	○	○	○
6-7 pm	○	○	○	○	○

15. In SPRING (SEPTEMBER TO NOVEMBER) , what times of the day would you usually be outside, and how long would you be outside for on a typical NON-WORKING DAY?

Please mark the corresponding circle - only one per line.

	Never	<15min	15-29min	30-44min	45-60min
5-6 am	○	○	○	○	○
6-7 am	○	○	○	○	○
7-8 am	○	○	○	○	○
8-9 am	○	○	○	○	○
9-10 am	○	○	○	○	○
10-11 am	○	○	○	○	○
11-12 pm	○	○	○	○	○
12-1 pm	○	○	○	○	○
1-2 pm	○	○	○	○	○
2-3 pm	○	○	○	○	○
3-4 pm	○	○	○	○	○
4-5 pm	○	○	○	○	○
5-6 pm	○	○	○	○	○
6-7 pm	○	○	○	○	○

Thanks for your time!

The Seasonal D Research Team
